# Microwave Pretreatment-Induced Significant Nutrient and Metabolite Changes in Sea Cucumber *Apostichopus japonicus*

**DOI:** 10.3390/md23060249

**Published:** 2025-06-11

**Authors:** Dairong Song, Fang Liu, Airong Jia, Xue Liu, Tingting Cui, Hui Zhang, Miansong Zhang

**Affiliations:** 1 Biology Institute, Qilu University of Technology (Shandong Academy of Sciences), Jinan 250103, China; songdairong2021@163.com (D.S.); liu1585835253@163.com (F.L.); jiaar@sdas.org (A.J.); liuxue@sdas.org (X.L.); tingtingcui@sdas.org (T.C.); zh000406@163.com (H.Z.); 2 Fujian Ocean Innovation Center, Xiamen 361102, China

**Keywords:** *Apostichopus japonicus*, microwave pretreatment, metabolite profile, non-targeted metabolomics analysis

## Abstract

The body wall of sea cucumbers is rich in nutrients and small-molecule metabolites; however, traditional hot water pretreatment often leads to nutrient loss. To optimise processing techniques, this study compared the effects of microwave pretreatment and conventional hot water pretreatment on nutrient retention and metabolite profiles. Untreated sea cucumber body wall samples served as controls. The samples were subjected to microwave pretreatment (4 W/g, 12 min) or hot water pretreatment (100 °C, 10 min). Nutrient retention rates and metabolite variations were systematically analysed. Microwave pretreatment demonstrated superior retention of protein (96%), crude fat (92%), total sugar (55%), and saponins (40%). It also promotes the accumulation of small-molecule metabolites, including spermidine, tagatose, and melatonin. Notably, the lysine and methionine retention rates were enhanced by 10-fold and 12-fold, respectively, while the vitamin A, vitamin B3, and melatonin retention increased by 2.4-fold, 2-fold, and 3-fold, significantly outperforming traditional pretreatment. These findings highlight the potential of microwave pretreatment as an efficient alternative to conventional methods for preserving the nutritional and functional components of sea cucumbers.

## 1. Introduction

Sea cucumbers, classified as echinoderms belonging to the Holothuroidea class, encompass a diverse array of species. Presently, approximately 1250 species of sea cucumbers have been identified, with roughly 70 recognised as edible varieties [1,2,3,4]. *Apostichopus japonicus* is the primary sea cucumber species used to produce sea cucumber products in Japan, Korea, China, Russia, and other nations. Furthermore, it is a sea cucumber variety with exceptional nutritional attributes [5]. From a nutritional perspective, sea cucumbers are considered highly nutritious seafood due to their comprehensive profile of dietary components, such as collagen, fucosylated chondroitin sulphate, and fucoidan sulphate. *Apostichopus japonicus* is frequently used as a primary nutritional supplement for patients, especially those with haemorrhages [6].

Furthermore, sea cucumber body walls are nutritionally and medicinally valuable due to their bioactive compounds. These include collagen, polysaccharides, lipids, amino acids, fatty acids, vitamins, minerals, saponins, carotenoids, chondroitin sulphate, and other bioactive substances [7]. These components have different biological activities that support human health, such as boosting immunity, preventing ageing, improving cognitive function, reducing blood pressure and cholesterol, and possessing antibacterial and antioxidant properties [7].

Sea cucumbers are highly susceptible to autolysis after harvesting due to the increase in endogenous protease activity in the body wall [8]. Therefore, measures should be taken immediately after harvesting to prevent autolysis. Traditional boiling water pretreatment (100 °C for 10 min) effectively inhibits the activity of sea cucumber endogenous protease. This traditional pretreatment consumes energy and produces large amounts of organic-rich wastewater, depleting sea cucumber nutrients and biologically active constituents. Yin, et al. [9] researched the changes in sea cucumber’s nutritional composition after boiling. Studies have found that traditional boiling pre-processing results in the loss of nutrients and potent ingredients, including 50% of minerals, 11.5% of proteins, 33% of carbohydrates, and 41% of sea cucumber saponins [9]. Subsequent analysis using LC-MS and GC-MS revealed the loss of many secondary metabolites, including the degradation of specific protein components into amino acids and a decrease in the content of some fatty acids, nucleic acids, organic acids, vitamins, and other nutrients. It was found that 100% of the spermidine was lost during the pretreatment process [9]. Therefore, it is necessary to identify an alternative to the conventional boiling water pretreatment method for inhibiting endogenous enzyme activity in sea cucumbers while simultaneously reducing the quantity of boiling water discharged and minimising nutrient loss from sea cucumbers.

Microwave energy has proven to be a highly effective heat source in the food industry due to its ability to induce rapid internal heating through mechanisms such as dipole rotation and ionic conductance within the material [10]. The use of microwaves enables the concurrent heating of the internal and external regions of materials, thereby enhancing the overall heating efficiency. High temperatures can inactivate enzymes, and the rapid warming of the sea cucumber body wall by microwaves may quickly and effectively reduce endogenous enzyme activity in the sea cucumber body wall. Furthermore, microwave heating does not require the addition of any external water during the heating process [11], which prevents the loss of nutrients in sea cucumbers. Currently, there is a lack of research on the direct application of microwave technology for the treatment of fresh sea cucumbers. Accordingly, the microwave treatment conditions were established in this study to inhibit endogenous protease activity in sea cucumbers. Thereafter, the extent of microwave pretreatment in terms of nutrient retention and changes in small-molecule metabolites was investigated by comparing it with conventional pretreatment methods.

The purpose of this study is to compare the effects of traditional boiling and microwave processing on sea cucumber pretreatment, highlight the advantages of microwave pretreatment, and provide a theoretical basis for updating sea cucumber pretreatment processing methods.

## 2. Results and Discussion

### 2.1. Effect of Microwave Power and Microwave Time on the Dehydration of Frozen Sea Cucumber Body Wall

**Figure 1:**
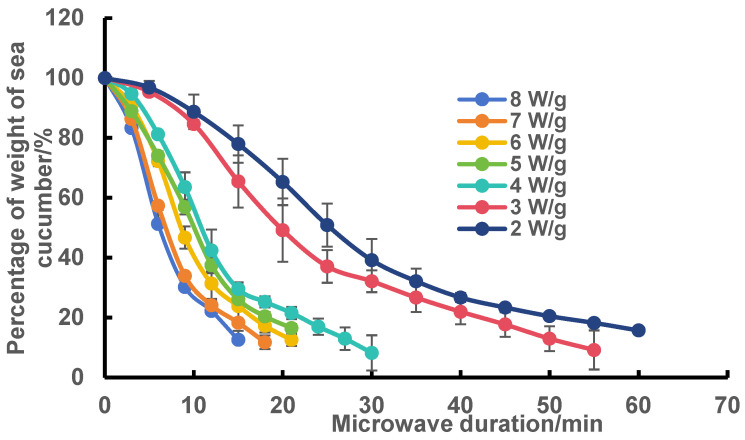
Dehydration curves of the frozen sea cucumber body wall under different microwave power conditions.

As shown in Figure 1, the dehydration rate of the sea cucumber body wall gradually increased with increasing microwave power. When the microwave power was 2 W/g and 3 W/g, the drying times were 60 min and 55 min, respectively, while the drying time was shortened to 15–30 min when the microwave power was increased to 4, 5, 6, 7, and 8 W/g. These observations are comparable to the results reported in the literature for different drying methods of other products [12]. Each drying curve could be divided into two stages: fast and slow dehydration periods. The dehydration curves were divided into two groups with 4 W/g as the dividing line because the pretreatment of sea cucumbers aims at partial dehydration rather than direct drying. Therefore, 4 W/g is the optimal choice.

### 2.2. Effect of Microwave Pretreatment on Endogenous Protease Activities in Frozen Sea Cucumber Body Wall

**Figure 2:**
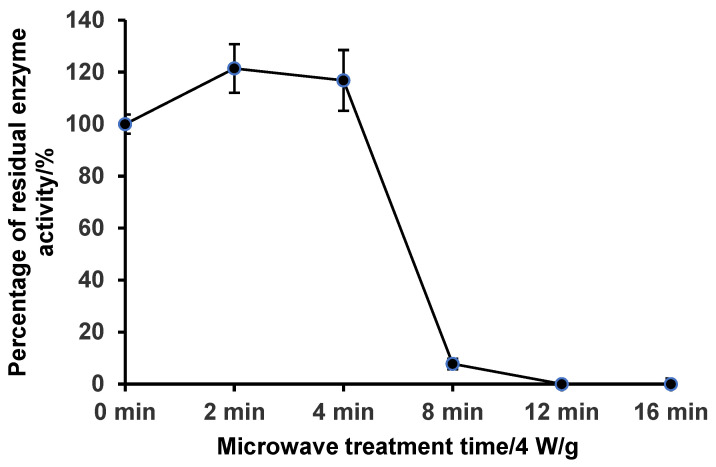
Residual enzyme activity of endogenous protease in the body wall of sea cucumber at different treatment times.

**Figure 3:**
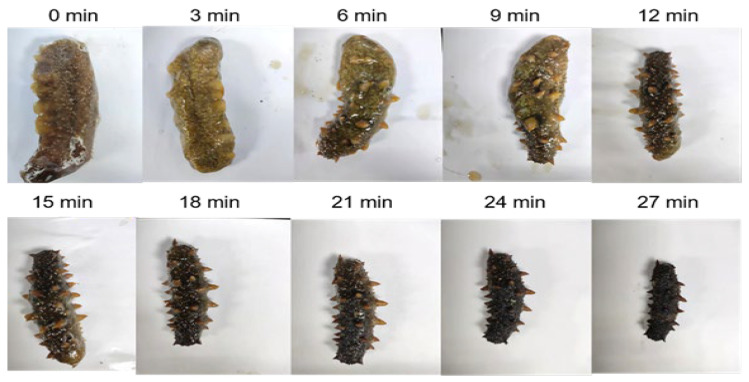
Microwave dehydration process of sea cucumber (4 W/g).

As shown in Figure 2, under the microwave intensity of 4 W/g, the endogenous protease enzyme activity in the body wall of frozen sea cucumber had a slight increase at the beginning of microwave thawing and then began to decrease after 2 min and was close to 0 after 12 min, and then the endogenous protease enzyme activity of sea cucumber remained 0 until 16 min. Figure 3 shows the shape changes of the sea cucumber at each time period under a microwave intensity of 4 W/g. After 12 min, the body wall of the sea cucumber was already in better shape for subsequent processing (Figure 3). Therefore, based on the residual enzyme activity and appearance, 4 W/g for 12 min was selected as the microwave pretreatment condition.

### 2.3. Comparison of Nutrient Contents in Sea Cucumber Body Walls with Different Pretreatments

The results of the one-way ANOVA analysis by SPSS software (Version 20.0, SPSS Inc., Chicago, IL, USA) revealed a significant enhancement in nutrient retention within the body wall of sea cucumbers when treated with MTW compared to that with BTW. This improvement was observed across various components, including total protein (96%), fat (92%), carbohydrate (55%), and saponin (40%) retention. However, no significant difference was observed in the total ash content (Table 1). A previous study by Heiss and Azadi [13] highlighted that the existing literature does not provide direct evidence substantiating that microwaves can harm the covalent or other chemical bonds within proteins present in food. Moreover, from an energy perspective, the photons emitted by microwaves possess insufficient energy to disrupt the chemical bonds in proteins. Microwaves may destroy the collagen structure and increase the amino acid content. Microwave treatment had no significant effect on the saponin content of sea cucumbers [14]. It is speculated that the effluent may cause saponin loss in sea cucumbers during microwave pretreatment with fresh sea cucumbers. Under the influence of microwave radiation, a retention rate of over 90% for fats was observed, which was primarily attributed to the stability of unsaturated fatty acids during microwave processing [15]. According to Iwaki et al. [16], carbohydrates, which are vital food constituents, undergo various transformations during microwave pretreatment. The prevailing chemical reactions observed include the caramelisation of sugars and the Maillard reaction, which can result in the degradation or depletion of carbohydrates.

### 2.4. Differences in Metabolites Between MTW, BTW, and UTW Based on GC-MS Analysis

**Figure 4:**
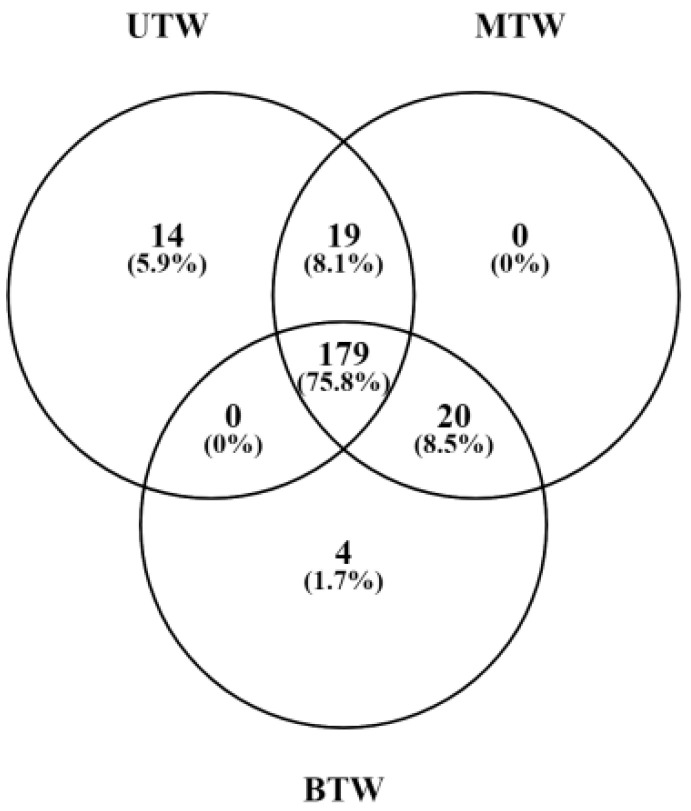
Venn diagram of the differential distribution and proportion of small-molecule metabolites detected by GC-MS in UTW, BTW, and MTW.

A Venn diagram illustrating the number of metabolites present in each sample was generated (Figure 4). Specifically, UTW contained 212 small-molecule metabolites, MTW contained 218 small-molecule metabolites, and BTW contained 203 small-molecule metabolites. Comparing the metabolite compositions between MTW and BTW, we found that 199 metabolite compositions were shared between the two pretreatments. Among these shared metabolites, 19 were exclusively detected in MTW, and four were exclusively detected in BTW. Spermidine, tagatose, and guanidinoacetic acid were identified in MTW but not in BTW. Notably, prior research has revealed the various functions of spermidine, including its ability to enhance cardiovascular function, delay the onset of Alzheimer’s disease, and its potential as a therapeutic agent for anti-psychotic disorders [17]. Studies have shown that tagatose, used in the food and pharmaceutical industries, exhibits properties such as hyperglycaemia inhibition, enhancement of intestinal flora, and non-induction of dental caries [18]. Guanidinoacetic acid, a precursor of creatine biosynthesis, plays a role in the regulation of energy metabolism in organisms. Furthermore, guanidinoacetic acid possesses anti-inflammatory and antioxidant capabilities, which can facilitate muscle growth [19].

### 2.5. Differences in Metabolites Between MTW, BTW, and UTW Based on LC-MS Analysis

**Figure 5:**
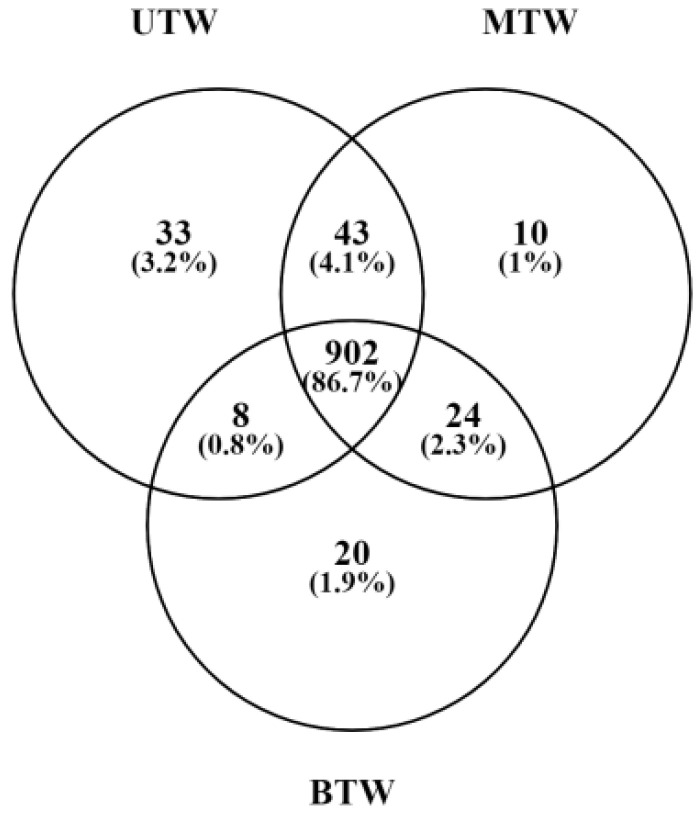
Venn diagram showing the differential distribution and proportion of small-molecule metabolites detected by LC-MS in UTW, BTW, and MTW.

Hundreds of small-molecule metabolites (986) were detected in the UTW, while 979 were detected in the MTW. The total number of small-molecule metabolites identified in BTW was 954 (Figure 5). A total of 945 metabolites were shared between the two pretreatments, UTW and MTW. Among these, 41 metabolites were exclusively detected in UTW, while 34 were exclusively detected in MTW. Lysophospholipids were detected in MTW, but no lysophospholipids were detected in UTW or BTW. Lysophospholipids are intermediate products of glycerophospholipid metabolism, which refers to phospholipids formed by the removal of one fatty acid moiety from phospholipids. These findings suggest that microwave pretreatment may facilitate the conversion of lysophospholipids [20]. A comparison of the metabolite compositions detected in both MTW and BTW pretreatments revealed that they had 926 shared metabolite compositions. Among these, 53 metabolites were exclusively detected in MTW, while 28 metabolites were solely detected in BTW. Previous research has identified spermidine as a natural polyamine with a high content in neuronal cells. Spermidine plays a crucial role in regulating synaptic plasticity, promoting autophagy, and mitigating oxidative stress in the nervous system [21].

### 2.6. Significant Differences of the Metabolites in Sea Cucumber Body Walls with Different Pretreatment by GC-MS Analysis

By analysing the relative ratio of metabolites in MTW to UTW using GC-MS, 17 metabolites with differential distribution were identified. These included eight amino acids and their derivatives, one vitamin, four sugars, two organic acids, and two fatty acids, as shown in Table 2. Notably, the levels of linoleic acid and arachidonic acid, which are polyunsaturated fatty acids, were higher in MTW than in UTW. Microwave pretreatment may convert a portion of the fat in sea cucumber body walls into fatty acids [22]. Several studies have demonstrated the critical role of polyunsaturated fatty acids in promoting cerebral development in infants, enhancing the regulatory function of the immune system, and preventing various diseases [23].

Based on the MTW to BTW ratio, GC-MS analysis revealed 19 metabolites with differential distribution, comprising 11 amino acids and their derivatives, one nucleic acid and its derivatives, two organic acids, and five sugars ( Table 3). The comparison of MTW to BTW ratios indicated higher amino acid content in MTW relative to BTW, with methionine and lysine showing notably elevated retention levels at 12-fold and 10-fold, respectively. This may be attributed to the detrimental impact of microwave radiation on collagen, leading to increased amino acid levels [14]. Notably, methionine and lysine are essential constituents of sea cucumber body wall proteins and represent amino acids that the human body cannot endogenously synthesise. Methionine exhibits hepatoprotective properties against hepatic sclerosis, fatty liver disease, and various forms of acute, chronic, and viral hepatitis [24]. In contrast, lysine plays a crucial role in brain development, fat metabolism, immune enhancement, and antiviral activities, among other functions [25].

Compared to BTW, MTW exhibited higher retention of organic acids, namely, creatine and guanidinoacetic acid, with retention levels threefold and ninefold greater, respectively. Guanidinoacetic acid (GAA) is a significant precursor of creatine synthesis in vertebrates. Functionally, creatine enhances the aerobic energy supply in the human body, elevates athletic performance, and facilitates the reduction of exercise-induced fatigue [26]. In contrast to hot water pretreatment of the sea cucumber body wall (BTW), microwave pretreatment displayed superior retention of glycans, particularly tagatose, with an augmented retention rate of fivefold. Tagatose exhibits a range of beneficial functions, such as reducing blood glucose levels in diabetic individuals, mitigating obesity, improving human intestinal flora, and exhibiting anti-caries effects and antioxidant activities [27].

### 2.7. Significant Differences of the Metabolites in Sea Cucumber Body Walls with Different Pretreatment by LC-MS Analysis

Through differential metabolite analysis of LC-MS data, a total of 54 metabolites exhibiting differential distribution were identified, encompassing 30 glycerophospholipids, one organic acid, three amino acids, three nucleic acids, and their derivatives, three fatty acids, two vitamins, nine glycolipids, various other nutrients (as delineated in Table 4). When comparing microwave pretreatment with traditional pretreatment, the former showcased elevated levels of fatty acids, certain glycerophospholipids, and heightened concentrations of the unsaturated fatty acid linolenic acid. This phenomenon could be attributed to the potential breakdown of certain fats induced by microwave pretreatment [28].

Compared to UTW, MTW (microwave pretreatment) exhibited a reduction in amino acids, succinic acid, most nucleotides, and vitamin B5. The observed decrease in uracil and deoxythymine nucleotides may be attributed to the rupture of DNA and RNA hydrogen bonds within sea cucumber body wall cells, which could potentially result from the oxidative stress induced by microwave pretreatment of said cells [29].

Compared to UTW, MTW (microwave pretreatment) led to increased levels of levodopa. Levodopa primarily functions to support nerve health, sustain regular muscle function, and exhibit protective and rehabilitative properties on limb and muscle activity in individuals with Parkinson’s disease [30].

Compared to hot water pretreatment of the sea cucumber body wall, microwave pretreatment exhibited a higher retention of L-carnitine, with a retention rate increase of 1.3 times. L-carnitine facilitates intestinal development, enhances the digestive and absorptive capacities of the human body, and possesses immune-boosting properties. Moreover, it can reduce hepatic fat deposition, promote excretory function, and regulate glucose metabolism [31].

Based on the analysis of LC-MS data for differential metabolites, 60 metabolites were found to be differentially distributed. The detected metabolites comprised 41 glycerophospholipids, three amino acids, two nucleic acids and their derivatives, seven fatty acids, two vitamins, two glycolipids, and other substances (Table 5). Comparatively, MTW (microwave pretreatment) demonstrated a lesser impact on fat degradation than BTW (hot water pretreatment), resulting in a higher retention of fat, which aligns with the findings presented in Table 1, demonstrating increased fat retention in MTW compared to that in BTW. Additionally, when comparing MTW with BTW, there was a noticeable retention of amino acids, nucleotides, vitamin A, and vitamin B3 (refer to Table 5). Vitamin A plays a significant role in preventing glaucoma and improving visual function in patients with glaucoma [32]. Vitamin B3, also known as niacin, possesses preventive and therapeutic properties against skin diseases and deficiencies related to similar vitamins. It exerts a vasodilatory effect, making it useful in pretreatment conditions, such as peripheral nerve spasms and arteriosclerosis [33]. Furthermore, microwave pretreatment resulted in significantly better melatonin retention than hot water pretreatment, exhibiting a 2.8-fold increase in retention. Melatonin is known to enhance the growth and development of plants and animals and improve the quality of sleep in humans [34].

## 3. Materials and Methods

### 3.1. Reagents

D-(+)-Galactose was purchased from Sigma-Aldrich Co., (St. Louis, MO, USA). L-2-Chloro-L-phenylalanine was procured from Shanghai Hengbai Biotechnology Company (Shanghai, China). BSTFA ( 1% *v*/*v*) was purchased from REGIS Technologies (Morton Grove, IL, USA). Fatty acid methyl esters (FAMEs) were purchased from Sigma-Aldrich (Shanghai, China) trading Co., (Shanghai, China). Deionised water was obtained from a laboratory water purifier which is produced by Chongqing Molecular Water Treatment Equipment Co., Ltd. (Chongqing, China). Ginsenoside Re was obtained from Shanghai Yuanye Biological Technology Co., Ltd. (Shanghai, China). All other chemicals and reagents purchased from Sinopharm Chemical Reagent Co. Ltd. (Beijing, China) were of analytical grade.

### 3.2. Sea Cucumber Materials

Live adult farmed sea cucumbers (*Apostichopus japonicus*, 120.0 ± 10.0 g) were purchased from the farm of Shandong Homey Aquatic Development Co., Ltd. (Weihai, China) in October 2023. After evisceration, the body walls were stored separately in a −80 °C freezer for further use.

### 3.3. Plotting of Microwave Dehydration Curves

Frozen sea cucumber body walls with a 60.0 ± 5.0 g mass were selected for sea cucumber dehydration tests. The microwave intensities of 2 W/g, 3 W/g, 4 W/g, 5 W/g, 6 W/g, 7 W/g and 8 W/g were used by an experimental microwave oven (HWL08-A) which is produced by Hangzhou Quanbo Biotechnology Co., Ltd. (Hangzhou, China), and the recordings were started from the frozen sea cucumber body wall, and the experiments were stopped at the time when the relative moisture content of the body wall of the sea cucumber reached (12 ± 1)%. Dehydration curves for different microwave intensities were plotted using the weight percentage of sea cucumbers as the vertical coordinate and microwave duration as the horizontal coordinate. The experiments were repeated three times, and the results were expressed as the mean ± standard deviation. The optimum microwave intensity was selected based on the dehydration rate.

### 3.4. Effect of Microwave Pretreatment on Endogenous Protease Activities in Sea Cucumber Body Wall

The impact of microwave treatment time on sea cucumber endogenous protease activity was investigated at the optimum microwave intensity. The investigations determined the ideal microwave processing density and processing duration based on the sea cucumber endogenous protease activity and shape change of the sea cucumber body wall.

### 3.5. Sample Preparation for Compositional Analysis and Metabolomics Analysis

Six frozen sea cucumber body walls were randomly selected, treated with microwaves at a density of 4 W/g for 12 min, and freeze-dried (Microwave-treated Sea cucumber body walls, MTW, *n* = 6). Another six frozen sea cucumber body walls were randomly selected, treated with boiling water for 10 min, and freeze-dried (Boling water-treated sea cucumber body walls, BTW; *n* = 6). Six untreated fresh sea cucumber body walls were selected and freeze-dried (Untreated sea cucumber body walls, UTW, *n* = 6). The lyophilised samples were stored at −80 °C until analysis.

### 3.6. Determination of Proximate Composition

The moisture content was determined by oven drying using the AOAC Method 950.46 [35]. Crude protein content was analysed by Kjeldahl nitrogen determination according to AOAC Method 981.10. The ash content was determined through mineralisation, following the guidelines provided in AOAC 920.153. The Soxhlet extraction method was used to determine the crude fat content [36]. The phenol sulfuric acid method was used to determine the total sugar content [37].

### 3.7. Determination of Total Saponin Content

A quantitative analysis of the total saponin content was conducted utilising an approach derived from the methodology proposed by Yin, Jia, Heimann, Zhang, Liu, Zhang, and Liu [9]. Crushed freeze-dried sea cucumbers were extracted with 20% ethanol by ultrasonication, dried, re-solubilised with water, adsorbed, and resolved using D101 macroporous columns. The 70% ethanol eluate was collected and dried for further analysis. The specific steps for the subsequent determination were: the dried extracts were dissolved in 0.2 mL of 5% vanillin and mixed with 0.8 mL of perchloric acid. After a 10-min water bath treatment at 60 °C and subsequent cooling, 5.0 mL of acetic acid was added. The resulting colour development was quantified at 560 nm using a microplate reader (Tecan Infinite 200 pro, TECAN Group, Austria, Switzerland). A standard curve was generated using increasing concentrations of ginsenoside Re standard solutions prepared by diluting a ginsenoside Re stock solution (0.2 mg/mL). The total saponin content of the samples was calculated using a standard curve.

### 3.8. Proteinase Activity and the Residual Enzyme Activity

Non-specific proteinase activities were assayed using casein as a substrate, as described by Yu-Xin et al. [38], with slight modifications. Next, 1 mL of sea cucumber body wall tissue extract was added to a 5 mL centrifuge tube preheated for 5 min in a 40 °C water bath. Three parallels were prepared for each group of samples, and a blank control was also prepared. Additionally, 2 mL of 1.0% casein, which had been preheated for 5 min in a 40 °C water bath, was added. The reaction was conducted at 40 °C for 30 min. Finally, trichloroacetic acid was added to terminate the reaction. Trichloroacetic acid should be added to the blank tube first to stop the enzymatic reaction before adding the substrate. Subsequently, 1.0 mL of the supernatant was mixed with 5 mL of 0.55 moL/L Na_2_CO_3_, and 1.0 mL of Folinophenol reagent B was added and shaken well. Colour development was conducted at 40 °C for 15 min, and the absorbance values of the resulting supernatants were measured at 680 nm using a UV 2100 spectrophotometre (Unico Instrument Co., Ltd., Shanghai, China). One enzyme unit was defined as the enzyme required to produce 1 μg of amino acid per mL of digest per min. Under the reaction conditions, the amount of enzyme necessary to generate 1 μg of amino acid per minute per millilitre of enzyme solution was considered as one enzyme unit.

Residual enzyme activity refers to the amount of enzymatic activity that remains in the sample after treatment has been performed. It is often expressed as a percentage of the initial enzyme activity, which is the activity percentage before treatment.

$$\mathrm{Residual}\;\mathrm{enzyme}\;\mathrm{activity}\;(\%)=\frac{A_{1}}{A_{0}}\times 100$$

*A*_1_, whichis the final enzyme activity, the amount of enzyme activity remaining after the treatment; and *A*_0_ is the initial enzyme activity, the amount of enzyme activity present before the treatment.

### 3.9. GC-MS Analysis of UTW, BTW, and MTW

#### 3.9.1. Sample Preparation for GC-MS Analysis

Sample preparation followed the procedure described in Section 3.5, resulting in the generation of UTW, BTW, and MTW samples. Subsequently, metabolite extraction was conducted following the protocol established by Want et al. [39]. Specifically, sea cucumber sample powder weighing 50 ± 1 mg was transferred into a 2 mL EP tube, followed by methanol: chloroform extraction solution in a volume ratio of 3:1, totalling 450 μL. L-2-chlorophenylalanine (10 μL) was then added to the EP tube, and the solution was thoroughly mixed for 30 s using a vortexer. Ceramic beads were subsequently introduced to the sample solution, which was milled at a rate of 45 Hz for 4 min using a grinder and then cooled at 0 °C for 5 min. The sea cucumber sample mixture was centrifuged at 4 °C and 12,000× *g* for 15 min using a freezing centrifuge. Quality control (QC) samples were prepared by pooling 60 μL aliquots from each sample group. Each sample was dried using a vacuum concentrator. Extracts: To the metabolites of the dried sea cucumber samples, 60 μL of methoxylated ammonium salt reagent dissolved in 20 mg/mL pyridine was added accurately, and the mixture was mixed well and then placed in an oven for 30 min at 80 °C. To each group of sea cucumber samples, 80 μL of BSTFA reagent containing 1% TMCS was added, and the mixture was placed in a 70 °C thermostat and incubated for 1.5 h. The samples were cooled to room temperature, and 5 μL of BSTFA reagent containing 1% TMCS was added to the mixture. The mixture was then incubated at 70 °C for 1.5 h. After cooling to room temperature, 5 μL of FAMEs dissolved in chloroform was added to the mixed samples, which were stored in an ultra-low-temperature refrigerator at −80 °C for further analysis.

#### 3.9.2. GC-MS Analysis

The derivatised samples were analysed using an Agilent 7890 B Gas Chromatograph (Santa Clara, CA, USA) coupled with an Agilent DB-5MS. The liquid-phase experiments were separated on a capillary column (30 m × 0.25 mm × 0.25 μm, Agilent J&W, Folsom, CA, USA). Helium gas (>99.999%) was used as the carrier gas at a constant flow rate of 3 mL/min. The injector temperature was maintained at 260 °C, and a 1 μL injection volume was used in splitless mode. The initial oven temperature was set at 50 °C for 1 min, followed by a linear increase to 310 °C at a rate of 20 °C/min for 6 min. A quadrupole mass spectrometer was used for mass spectrometry analysis. The temperatures of the mass spectrometry quadrupole and the ion source (electron impact) were set at 150 °C and 250 °C, respectively. The collision energy applied was −70 eV. Mass spectrometry data were acquired in the full scan mode (*m*/*z* 50–500) with a solvent delay of 4.78 min. Periodic injection of mass controls was performed throughout the analysis to evaluate the reproducibility.

#### 3.9.3. Metabolites Identification

Peak extraction, baseline correction, deconvolution, peak integration, and peak alignment were performed to analyse the mass spectrometry data using Chroma TOF (version 4.34, LECO, St. Joseph, MI, USA) software. The metabolites were qualitatively identified based on mass spectral match, retention time index match, etc., using the ChemStation (version E.02.02.1431, Agilent, Santa Clara, CA, USA) software.

### 3.10. LC−MS Analysis of UTW, BTW, and MTW

#### 3.10.1. Sample Preparation for LC-MS Analysis

Each pulverised sample (100 mg) was freeze-dried, placed in an EP tube, and mixed with 500 μL of 80% methanol aqueous solution (containing 0.1% formic acid) by vortexing. The mixture was then allowed to stand in an ice bath for 5 min, centrifuged for 10 min at 15,000× *g* and 4 °C, and a certain amount of supernatant was collected and diluted with methanol to a concentration of 53%. A certain amount of supernatant was taken and diluted with mass spectrometry grade to 53% methanol content, which was then placed in a centrifuge tube and processed by freezing centrifugation at 15,000× *g* at 4 °C for 10 min. The precipitate was discarded, and the supernatant was collected for further analysis. The sample solution was analysed using LC-MS [40]. Mixtures of equal volumes of the solutions from the three pretreatments of the powdered sea cucumber samples were used as QC samples. Blank samples: The concentration of methanol aqueous solution used for the experiment was changed to 53% methanol aqueous solution instead of the experimental samples, and the pretreatment experimental process was the same as that of the sea cucumber samples.

#### 3.10.2. LC-MS Analysis

An ACQUITY UHPLC system (Waters Corporation, Milford, MA, USA) was combined with an AB SCIEX Triple TOF 5600 system (AB SCIEX, Framingham, MA, USA) to analyse the metabolites in the ESI positive and negative ion modes using a Hypesil Gold column C18 column. The binary gradient elution system consisted of (A) water (containing 0.1% formic acid, *v*/*v*) and (B) methanol solution. Separation: 2% B in 0 min, 2% B in 0–1.5 min, 100% B, 1.5–12 min, hold at 100% B for 2 min, hold at 100–2% B for 14–14.1 min. 100–2% B and hold at 2% B for 14.1–17.0 min. The flow rate was 0.4 mL/min and the column temperature was 45 °C. All samples were analysed at a flow rate of 0.4 mL/min. All samples were stored at 4 °C during the analysis. The injection volume was 5 µL for each sample. Data acquisition was performed in the full scan mode (*m*/*z* 70–1000) combined with the IDA mode. The mass spectrometry parameters were as follows: ion source temperature 550 °C (+) and 550 °C (−); ion spray voltage 5500 V (+) and 5500 V (−). Voltages 5500 V (+) and 4500 V (−), curtain gas 35 PSI, and dust removal potentials 100 V (+) and −50 V (−). Collision energies of 10 eV (+) and −10 eV (−) and interface heater temperatures of 550 °C (+) and 600 °C (−). For the IDA analysis, the *m*/*z* range was set to 50–1000, and the collision energy was set to 30 eV. The energy was 30 eV. The QCs were injected periodically throughout the analytical run. To assess reproducibility, we used the

#### 3.10.3. Metabolite Identification

The raw data files generated by the assay were imported into Progenesis QI 2.0 version software (Waters Corporation, Milford, MA, USA) for initial data processing. This involved brief screening based on the retention time and mass-to-charge ratio. Subsequently, the peaks from different samples were aligned with a retention time deviation of 0.2 min and a mass deviation of 5 parts per million (ppm) to enhance identification precision. The mass deviation was further refined to 5 ppm, the signal intensity deviation was set to 30%, and the signal-to-noise ratio was 3. Moreover, the minimum signal intensity was established at 100,000, and the information derived from the summed ions was used to predict the molecular formulas. The combined positive and negative data sets were imported into the SIMCA software package (version 14.1, Umetrics, Umeå, Sweden). Background ions were eliminated using blank samples, and the quantitative results were normalised to yield the outcomes of metabolite identification and quantitative analysis in the experimental samples.

### 3.11. Statistical Analysis

Simca 14.1 software was employed to conduct Principal Component Analysis (PCA) and Orthogonal Partial Least Squares Discriminant Analysis (OPLS-DA) to visually depict the metabolic differences among experimental groups. The data were subjected to unit variance (UV) scaling and mean post-centre visualisation. The t2 regions of Hotelling, represented as ellipses in the fractional plots of the models, were used to establish 95% confidence intervals for the changes in the model. Variable Importance in Prediction (VIP) was used to rank the overall contribution of each variable to the OPLS-DA model, with variables having a VIP value greater than 1 being considered potentially associated with group discrimination [41]. This study employed 200 response rankings to avoid potential errors [42]. Statistically significant thresholds for Variable Importance in Prediction (VIP) values (>1) and *p*-values (<0.05) were determined using *t*-tests conducted on normalised peak areas.

## 4. Conclusions

In this study, the conventional nutrients and metabolites in the microwave-treated sea cucumber body wall (MTW), hot water-treated sea cucumber body wall (BTW), and untreated sea cucumber body wall (UTW) were comparatively evaluated. Microwave pretreatment enhanced the retention of proteins by up to 96%, fats by up to 92%, carbohydrates by up to 55%, and saponins by up to 40% compared to hot water pretreatment. Both microwave and hot water pretreatments led to a significant reduction in ash content. Through LC-MS and GC-MS-based metabolite analysis, spermidine, tagatose, and guanidino acetic acid were effectively preserved by microwave pretreatment but lost during hot water pretreatment. Moreover, compared to the untreated sea cucumber body wall, microwave pretreatment increased the levels of essential fatty acids, such as linoleic acid, linolenic acid, and arachidonic acid, and other nutrients like levodopa.

Furthermore, microwave pretreatment exhibited higher retention rates of amino acids, nucleotides, organic acids, and vitamins than hot water pretreatment. Notably, the retention rates of lysine and methionine in the microwave-treated samples increased by 10 times and 12 times, respectively. Creatine retention increased 3.4 times, vitamin A 2.4 times, vitamin B3 approximately 2 times, and melatonin 3 times. These findings suggest that microwave pretreatment, instead of traditional hot water pretreatment, is beneficial for preserving nutrients and metabolites in sea cucumber body walls. This study provides valuable insights into the development of new sea cucumber products and functional foods.

## Figures and Tables

**Table 1:** Ash, total protein, fat, carbohydrate, and saponin contents in untreated sea cucumber body walls (UTW), microwave-treated sea cucumber body walls (MTW), and Boiling water-treated sea cucumber body walls (BTW).

Compounds (Dry Weight)	UTW (4.16 ± 0.34 g)	MTW (3.14 ± 0.18 g)	BTW (1.98 ± 0.42 g)
Protein			
mg/g (DW)	551.7 ± 16.6 ^a^	707.0 ± 14.0 ^b^	739.0 ± 13.0 ^c^
Total protein (mg)	2295.1 ± 187.6 ^b^	2220.0 ± 127.3 ^b^	1463.0 ± 310.4 ^a^
Compared with the untreated group (%)	--	96.73	63.75
Fat			
mg/g (DW)	37.62 ± 7.52 ^a^	46.00 ± 2.51 ^b^	31.84 ± 7.50 ^a^
Total fat (mg)	156.50 ± 31.28 ^b^	144.44 ± 7.88 ^b^	63.04 ± 14.85 ^a^
Compared with the untreated group (%)	--	92.29	40.26
Ash			
mg/g (DW)	37.22 ± 0.27 ^c^	17.49 ± 0.17 ^a^	26.28 ± 0.35 ^b^
Total ash (mg)	154.84 ± 1.12 ^b^	54.91 ± 0.53 ^a^	52.03 ± 0.69 ^a^
Compared with the untreated group (%)	--	35.46	33.60
Total sugar			
mg/g (DW)	106.16 ± 0.0211 ^c^	77.42 ± 0.0106 ^b^	67.58 ± 0.0023 ^a^
Total sugar (mg)	441.60 ± 0.0878 ^c^	243.10 ± 0.0616 ^b^	133.80 ± 0.0046 ^a^
Compared with the untreated group (%)	--	55.05	30.30
Total saponin			
mg/g (DW)	0.4595 ± 0.0132 ^c^	0.2445 ± 0.0327 ^a^	0.3533 ± 0.0251 ^b^
Total saponin (mg)	1.912 ± 0.0549 ^c^	0.7677 ± 0.1027 ^b^	0.6995 ± 0.0497 ^a^
Compared with the untreated group (%)		40.15	36.58

^a,b,c^ In each row, a different letter indicates significance between the two groups (*p* < 0.05).

**Table 2:** Differences in metabolites between MTW and UTW based on GC-MS analysis.

Metabolites	Quant. Mass	RT (min)	VIP	*p* Value	Relative Ratio (MTW/UTW)
amino acids					
aspartic acid	133	13.75	1.12	0.0126	0.46
leucine	131	10.52	1.17	0.0254	0.34
oxyproline	129	13.81	1.52	0.0013	0.32
threonine	119	12.02	1.47	0.0007	0.31
glutamic acid	147	14.95	1.55	0.0003	0.29
methionine	149	13.76	1.68	0.0104	0.29
glycine	75	10.99	1.92	0.0007	0.24
lysine	146	18.22	1.73	0.0015	0.21
vitamins					
pantothenic acid	219	18.91	1.17	0.0113	0.43
sugar					
tagatose	180	17.42	1.53	0.0370	0.47
erythrulose	120	12.71	2.58	0.0022	0.14
organic acids					
succinate	118	11.09	1.03	0.001	0.44
creatine	131	14.18	1.48	0.0003	0.31
fatty acids					
linoleate	280	21.00	1.37	0.0034	3.06
arachidonic acid	304	22.37	1.01	0.0006	2.13

**Table 3:** Differences in metabolites between MTW and BTW based on GC-MS analysis.

Metabolites	Quant. Mass	RT (min)	VIP	*p* Value	Relative Ratio (MTW/BTW)
amino acids					
methionine	149	13.76	2.91	0.0003	12.47
lysine	146	18.22	2.83	0	10.28
tyrosine	181	18.4	2.45	0	8.06
valine	117	9.73	1.78	0	4.61
aspartic acid	133	13.75	1.65	0.0011	4.53
phenylalanine	165	15.06	1.72	0.0001	4.50
proline	115	10.89	1.62	0.0015	4.13
isoleucine	131	10.8	1.68	0	4.02
glutamic acid	147	14.95	1.52	0.0001	3.78
threonine	119	12.02	1.37	0.0009	3.47
alanine	89	8.13	1.06	0.0001	2.48
nucleic acids and their derivatives					
n-alpha-Acetyl-L-guanine	174	15.91	1.31	0.0002	3.08
organic acids					
guanidineacetic acid	117	14.53	2.43	0.0005	9.36
creatine	131	14.18	1.37	0.0007	3.43
sugar					
fructose	180	17.75	2.70	0.0004	11.20
sorbets	180	17.66	2.37	0.0012	10.03
tagatose	180	17.42	1.61	0.03	5.68
isomaltose	342	26.08	1.58	0.0015	4.31
maltose	360	25.31	1.08	0.0026	2.27

**Table 4:** Differences in metabolites between MTW and UTW based on LC-MS analysis.

Metabolites	Quant. Mass	RT (min)	Ion Mode	VIP	*p* Value	Relative Ratio (MTW/UTW)
glycerophospholipids						
LPI 20:5	618	8.34	neg	2.09	0.0005	4.39
LPI 20:4	620	8.98	neg	2.29	0.0002	3.50
LPA 18:0	438	10.43	neg	1.7	0.0011	3.47
LPE 14:0	425	7.97	neg	1.53	0.0107	3.07
PC 0-20:2	547	9.68	pos	1.66	0.0081	2.42
LPE 18:3	475	8.38	neg	1.46	0.0076	2.38
PC 0-16:0-20:5	812	9.83	neg	1.69	0.004	2.36
LPA 20:5	456	8.37	neg	1.6	0.0016	2.34
LPA 20:4	458	8.89	neg	1.77	0.0038	2.33
LPC 0-18:3	503	9.68	pos	1.34	0.0026	2.21
LPE 18:2	477	8.84	pos	1.66	0.0069	2.20
LPE 16:2	449	8.04	neg	1.33	0.0486	2.12
LPE 18:2	477	8.83	neg	1.73	0.001	2.11
LPC 16:2	537	8.17	neg	1.28	0.0372	2.08
LPC 18:3	563	8.53	neg	1.38	0.0104	2.03
LPE 16:1	451	8.53	neg	1.48	0.0135	2.00
LPE 15:0	439	8.62	neg	1.57	0.0087	1.99
PC 24:2	617	10.52	pos	1.05	0.0124	1.89
LPC 20:2-SN1	547	9.95	Pos	1.43	0.0066	1.87
LPC 18:0-SN1	523	10.09	pos	1.33	0.019	1.81
LPA22:6	482	8.84	neg	1.27	0.037	1.8
LPE 0-15:1	423	9.11	neg	1.11	0.0384	1.78
LPC 15:0-SN1	481	8.81	pos	1.38	0.0193	1.78
LPC 14:0	467	8.93	neg	1.43	0.035	1.77
LPE 16:0	218	9.16	pos	1.37	0.0462	1.73
LPC 20:5	587	8.43	neg	1.12	0.0197	1.67
LPI 16:0	572	8.83	neg	1.31	0.0279	1.62
PC 0-15:0	963	8.56	pos	1.2	0.0343	1.60
LPC 16:0-SN1	495	9.42	pos	1.24	0.0462	1.54
PC 44:11	902	11.25	pos	1.4	0.0187	0.44
organic acids						
succinate	118	2.57	neg	1.32	0.0005	0.56
amino acids						
valine	214	2.03	pos	1.13	0.0005	0.49
proline	228	2.08	pos	1.34	0.0012	0.46
tyrosine	252	5.18	neg	2.28	0	0.10
nucleic acids and their derivatives						
uracil nucleotide	404	1.97	neg	1.51	0.0072	0.41
deoxythymidine nucleotide	322	5.27	neg	1.66	0.0084	0.39
deoxycytosine	307	3.44	neg	1.46	0.0099	0.35
fatty acids						
HFA 20:4/20:4	606	9.82	neg	1.48	0.0227	2.31
HFA 22:6/20:3	632	9.70	neg	1.23	0.0083	2.22
Linolenic (C18:2N6T)	280	8.18	pos	1.53	0.0063	2.12
vitamins						
vitamin D3	384	8.55	pos	1.25	0.0258	1.91
pantothenic acid	219	5.03	neg	1.51	0.0025	0.43
glycolipid						
LPS 19:0	539	11.03	neg	1.71	0.002	4.23
LPS 18:0	525	10.1	neg	1.15	0.0273	2.94
LPS 19:1	537	10.13	neg	1.74	0.0016	2.76
LPS 16:1	495	8.63	neg	1.44	0.0277	2.66
LPS 20:1	551	10.77	neg	1.75	0.001	2.64
LPS 20:4	545	8.89	neg	1.84	0.0027	2.56
LPS 22:4	573	10.77	neg	1.68	0.0009	2.53
LPS 18:1	523	9.6	neg	1.57	0.0145	2.35
LPS 21:1	565	11.33	neg	1.49	0.0055	2.28
LPS 22:5	571	9.15	neg	1.25	0.0429	1.76
others						
levodopa	197	5.98	neg	1.24	0.0072	2.75
carnitine	161	3.68	pos	1.74	0.0018	0.36
spermidine	145	1.07	pos	1.67	0.001	0.23

**Table 5:** Differences in metabolites between MTW and BTW based on LC-MS analysis.

Metabolites	Quant. Mass	RT (min)	Ion Mode	VIP	*p* Value	Relative Ratio (MTW/BTW)
glycerophospholipids						
galactosylceramide0-17:1-2:0	554	7.23	pos	1.56	0.0214	4.91
galactosylceramide0-15:1-2:0	504	6.7	pos	1.35	0.0278	3.03
LPE 0-15:1	423	9.11	neg	1.78	0.002	2.82
LPI 16:0	572	8.83	neg	1.85	0.0001	2.22
LPI 20:4	620	8.98	neg	1.1	0.0014	1.99
LPG 17:0	498	11.21	neg	1.01	0.0006	0.65
LPC 0-22:6	553	9.28	pos	1.21	0.0488	0.64
LPE 17:1	465	8.96	pos	1.12	0.047	0.63
LPC 0-16:1	479	9.29	pos	1.28	0.0066	0.61
LPC 20:3	591	9.36	neg	1.15	0.0039	0.59
LPC 17:1	553	9.16	neg	1.16	0.0273	0.58
LPC 20:0	582	9.52	neg	1.31	0.0204	0.58
LPC 20:1	595	10.47	neg	1.49	0.0085	0.56
LPC 22:6	613	8.86	neg	1.29	0.011	0.56
LPI 18:1	598	10.35	neg	1.13	0.0053	0.56
LPC 0-17:1	539	9.86	neg	1.66	0.0036	0.54
LPC 22:5	615	9.16	neg	1.07	0.0446	0.54
LPC 0-20:1	581	11.16	neg	1.18	0.0416	0.53
LPG18:1	510	10.51	neg	1.37	0.0022	0.52
LPC 21:1	609	10.93	neg	1.26	0.0199	0.51
LPE 18:4	473	7.94	neg	1.01	0.0287	0.51
LPC 0-20:5	573	8.91	neg	1.32	0.0172	0.49
LPE 20:3	503	9.16	neg	1.12	0.01	0.49
LPE 22:6	525	8.54	pos	1.47	0.0136	0.48
LPC 0-18:2	551	9.69	neg	1.32	0.0342	0.47
PG 20:5	530	8.67	neg	1.34	0.0105	0.47
LPC 22:1	623	11.39	neg	1.23	0.0133	0.46
LPC 0-19:2	565	10.08	neg	1.55	0.0084	0.41
Lysops 22:6	569	8.59	neg	1.83	0.0002	0.40
PC 0-22:6-2:0	641	9.48	neg	2	0.0001	0.39
PG 16:0	484	10.08	neg	1.8	0.0014	0.39
PL 0-18:5-3:0	513	8.63	pos	1.73	0.0017	0.36
LPA 18:0	438	10.61	neg	1.44	0.0275	0.36
LPC 0-20:4	575	9.42	neg	1.73	0.0046	0.34
LPE 20:5	499	8.3	neg	1.76	0	0.33
LPE 20:4	501	8.76	neg	1.95	0	0.30
LPC 0-17:2	537	8.5	neg	1.49	0.0046	0.28
PG 16:1	482	8.71	neg	1.34	0.0037	0.26
PG 20:2	536	11.2	neg	1.86	0.0004	0.24
LPE 22:5	527	8.98	neg	1.7	0.0003	0.22
amino acids						
phenylalanine	165	4.93	pos	1.65	0	3.23
L-Valyl proline	117	1.77	pos	1.54	0.0002	2.60
proline	115	1.41	pos	1.34	0.0083	2.20
nucleic acids and their derivatives						
deoxyadenosine monophosphate	331	2.01	neg	1.78	0.0002	3.23
uracil nucleotide	324	1.93	neg	1.35	0.0351	2.04
Vitamins						
vitamin A	286	9.26	pos	1.77	0.0057	2.43
nicotinic acid	123	1.86	pos	1.39	0.0006	1.91
fatty acids						
ST 28:2; 0; s	478	6.34	neg	1.32	0.0081	0.58
ST 27:1; 0; S	466	8.29	neg	1.08	0.0242	0.51
ST 29:1; 0; s	494	9.84	neg	1.08	0.0097	0.45
ST 24:1; 03; G;S	513	7.03	neg	1.62	0.0141	0.23
FAHFA 20:4/3:0	376	9.87	neg	1.54	0	0.22
FAHFA 22:6/3:0	400	9.77	neg	1.72	0.0012	0.21
FAHFA 20:5/3:0	374	9.39	neg	1.42	0	0.19
glycolipid						
LPS 22:6	569	8.77	neg	1.70	0.0000	0.37
LPS 22:1	579	6.95	neg	1.46	0.0328	0.18
others						
quercetin	302	4.92	neg	1.94	0.0001	3.05
melatonin	232	5.35	neg	1.48	0.0001	2.79
levodopa	197	5.98	neg	1.06	0.012	0.49

## Data Availability

Research data can be shared.
